# Enzyme immunoassays as a method for quantifying hair reproductive hormones in two felid species

**DOI:** 10.1093/conphys/cou044

**Published:** 2014-10-11

**Authors:** C. V. Terwissen, G. F. Mastromonaco, D. L. Murray

**Affiliations:** 1 Department of Biology, Trent University, Peterborough, ON, Canada K9J 7B8; 2 Toronto Zoo, 361A Old Finch Avenue, Scarborough, ON, Canada M1B 5K7

**Keywords:** Canada lynx (*Lynx canadensis*), domestic cat (*Felis catus*), estrogen, hair reproductive hormones, progesterone, testosterone

## Abstract

Non-invasive monitoring of wild felid reproductive states is important, given that many species reproduce poorly in captivity. Despite extensive work in faecal hormone analysis in felids, continued development of techniques is necessary, particularly with wild populations. In this study, our aims were as follows: (i) biochemical validation of enzyme immunoassays for estrogen, testosterone and progesterone in Canada lynx and domestic cat hair extracts; (ii) assessment of the use of hair reproductive hormones to differentiate between reproductive states (intact, estrus, pregnant and spayed/neutered), using domestic cats as a model; and (iii) assessment of the use of hair reproductive hormones to differentiate between age and sex, accounting for potential regional variability in wild lynx populations. Analysis of hair hormone levels showed prospective value in detecting pregnancy states, with pregnant domestic cats having higher levels of progesterone than spayed females. However, intact and pregnant cats did not differ in progesterone levels. Yet, two female domestic cats had higher levels of hair progesterone following a 38-day oral progestin treatment, perhaps providing a preliminary pharmacological validation of the method. Estrogen and testosterone did not differ statistically according to reproductive states of domestic cats, although intact males had higher levels of hair testosterone than neutered males. When we applied these techniques to lynx fur, we determined that hormone levels were not sufficiently precise to differentiate age classes. Hair reproductive hormone ratios differed between sexes, with the estrogen-to-progesterone ratio demonstrating the highest accuracy in differentiating males from females. Hair hormone levels differed regionally for wild lynx, indicating that spatial variability should be a consideration in wildlife hormone studies spanning large spatial scales. We conclude that use of hair hormone analysis by enzyme immunoassay may hold promise for differentiating sex in felids, but the technique will require further refinement and validation before it can be applied broadly and reliably.

## Abstract (precis)

Enzyme immunoassays were validated for the quantification of reproductive hormones (estrogen, testosterone, and progesterone) in the hair of two felid species. The method provides a tool to potentially determine sex and pregnancy states in wild populations, therefore improving the monitoring of felids that often have low reproductive success in captivity.

## Introduction

Wild cats are among the most endangered and vulnerable group of mammals in the world (Nowell and Jackson, 1996). Wild cats from the genus *Lynx* are monoestrous, seasonal breeders, with a short breeding season. Canada lynx (*Lynx canadensis*), which occupy the boreal forest of North America, breed from late February to early April (Quinn and Gardner, 1987; Fanson *et al.*, 2010b). It is understood that lynx productivity is highly correlated with the abundance of their primary prey, snowshoe hare (*Lepus americanus*), and during periods of high hare density, female lynx can reach sexual maturity at an accelerated rate (Quinn and Gardner, 1987). Lynx productivity is highest when snowshoe hare populations are at the peak of their 10 year numerical cycle and lowest when hare populations are at their nadir (Brand *et al.*, 1976). However, there is evidence that cyclic dynamics vary regionally in lynx, meaning that some populations can be numerically out of phase with other populations in other regions (Krebs *et al.*, 2013). It follows that such variation means that at a given time there should be regional differences in lynx reproductive states. Canada lynx are an important species for conservation, but the variability in their population dynamics provides challenges to population management. In fact, local lynx populations can be overexploited during cyclic lows when recruitment is minimal or non-existent (Bailey *et al.*, 1986; Poole, 2003), meaning that tools need to be developed to track their population status and trends effectively.

Hormone analysis can provide valuable information on the basic physiology and overall health of an animal, and specific hormones can be used to measure particular aspects of health (Sheriff *et al.*, 2011). Enzyme immunoassays (EIAs) are one method used for hormone analysis, but pharmacological validation must be carried out for the substrate of choice before the assay is widely adopted (Buchanan and Goldsmith, 2004). For example, studies of reproductive hormones in domestic cats (*Felis catus*) reveal that oral progestin increases baseline progesterone levels in faeces (Stewart *et al.*, 2010). To our knowledge, however, no similar validation has been attempted for hair reproductive hormone studies despite interest in the use of hair as a medium for hormone analysis.

Reproductive hormones have long been monitored through faecal analysis to provide information on reproductive function (Brown and Wildt, 1997; Dehnhard *et al.*, 2010). These hormones have been used to differentiate between age classes, because juveniles should produce lower levels of hormones than adults. Ratios of reproductive hormones generally serve to determine sex, given that both sexes produce testosterone, progesterone and estrogen (Rolland *et al.*, 2005). This framework has been applied to the determination of age and sex in the faeces of North Atlantic right whales (*Eubalaena glacialis*, Rolland *et al.*, 2005) and barred owls (*Strix varia*, Wasser and Hunt, 2005). It follows that as these same hormones are present in hair, then hair hormone analysis can be used to monitor similar reproductive parameters.

Hair hormone analysis has shown increasing popularity in ecology (MacBeth *et al.*, 2010; Brearley *et al.*, 2012; Bryan *et al.*, 2013), although to date the expansion from cortisol to additional steroid hormones, such as reproductive hormones, has been minimally exploited (Stalder and Kirschbaum, 2012). Hair provides a unique option for monitoring hormones, because concentrations in hair are representative of the level in the body during the period of hair growth, and thus, sampling can represent weeks to months of hormone production depending on the length of hair analysed (Sheriff *et al.*, 2011). This provides a longer-term assessment of status than is available using existing methods with blood (day of secretion; Genaro *et al.*, 2007) and faeces (lag of 24–48 h; Fanson *et al.*, 2011). It is thought that hair hormones are incorporated into the hair shaft from the follicle cells via the blood (Gow *et al.*, 2010; Russel *et al.*, 2011), which may provide an additional advantage because increased intra-individual changes in faecal analysis can be attributed to the composition of the gut bacteria (Palme, 2005; Dehnhard *et al.*, 2010). Reproductive hormones have been measured in the hair of rock hyrax (*Procavia capensis*, Koren *et al.*, 2006), cattle (*Bos taurus*, Gleixner and Meyer, 1997), humans (Yang *et al.*, 1998) and grizzly bears (*Ursus arctos*, Bryan *et al.*, 2013), but the present study is the first to quantify these hormones in a felid species.

The objectives of this study were as follows: (i) to validate EIAs biochemically for estrogen (E), testosterone (T) and progesterone (P) in Canada lynx and domestic cat hair extracts; (ii) to assess the use of hair reproductive hormones to differentiate between known reproductive states (intact, estrus, pregnant and spayed/neutered) using domestic cats as a model; and (iii) to assess the use of hair reproductive hormones to differentiate between age and sex, accounting for potential regional variability in wild lynx populations.

## Materials and methods

### Assay validation

Assay validation was conducted using methods previously described (see Terwissen *et al.*, 2013), with some exceptions. For precision, interassay coefficients of variation were evaluated using faecal extract controls, at 30 and 55–70% binding, loaded in duplicate on each plate. The efficiency of the extraction procedure was analysed using recovery of exogenous hormone at the following concentrations for lynx and domestic cats (E, 5 µl at 48 pg/μl; T, 5 µl at 32 pg/μl; and P, 10 µl at 6 pg/μl).

### Study animals

Wild hair samples were collected from lynx pelts at North American Fur Auctions (NAFA) in Toronto and Fur Harvesters Auction (FHA) in North Bay, Canada. All pelts were legally harvested by licensed trappers in the 2008–2009 trapping season and not tanned prior to collection. Peterborough Veterinary Clinic (PVC) in Peterborough, ON, Canada, and Cincinnati Zoo and Botanical Garden, in Cincinnati, OH, USA, provided domestic cat samples. Samples were collected with permission from the cat owners. Animals from PVC were privately owned pets, while those at Cincinnati Zoo were included in studies on felid reproductive biology (see Conforti *et al.*, 2013 for details). Two females were given oral progestin for 38 days as part of these studies. Samples (3 cm × 3 cm; cut down to the skin) were taken from a standardized location for each species (a hindlimb on lynx and a forelimb on domestic cats) using clean gloves and scissors. All samples were placed in envelopes and stored at room temperature until analysis.

#### Reproductive states: domestic cat

The owners provided the age (6 months to 12 years) and sex of the domestic cats (12 males, of which seven were neutered and five intact; and 27 females, of which 10 were spayed, five intact, five in behavioural estrus and seven pregnant). Individuals that had been spayed or neutered at least 1 year prior to EIA analysis were used to ensure a minimal period of hair growth since castration. Females were classified as in estrus using signs of behavioural estrus (i.e. rolling, peddling of hindfeet, vocalizing; Conforti *et al.*, 2013). Pregnancy was determined during spays performed by a licensed veterinarian or when kittens were reported by owners.

#### Age, sex and region: lynx

The age of lynx pelts was determined using pelt length (28 juveniles and 45 adults), with juveniles (or kits) having a pelt length <90.5 cm in the west and <81 cm in the east, while adults (or yearlings) had a pelt length >90.5 cm in the west and >82 cm in the east (Quinn and Gardner, 1984; Slough, 1996). The sex of lynx pelts (37 female and 36 male) was determined by amplifying the Y-chromosome-specific *Sry* locus and *Zfx* fragment on the X-chromosome (Woods *et al.*, 1999; Ortega *et al.*, 2004; Zigouris *et al.*, 2012). DNA extraction and amplification was performed as per Row *et al.* (2012), and allele sizes were scored manually with Genemarker version 1.7 (Softgenetics, State College, PA, USA). Lynx samples were classified into region by trap line location (37 east, in Quebec, Ontario and Manitoba; and 36 west, in Yukon and Alaska).

### Hair hormone analysis

Hair samples were extracted using methods previously described; see Terwissen *et al.* (2013). First, hair samples were washed with 100% methanol for 20 s using a spray bottle. Dried samples weighing between 10.4 and 68.0 mg were cut into 0.5 cm pieces into glass scintillation vials. To this, 100% methanol was added proportionally for 0.01 g/ml and extracted for 24 h on a rotator (Argos RotoFlex R2000, Elgin, IL, USA). Following centrifugation (10 min at 2629 *g*) 750 or 1500 µl of the extracted solution was transferred to a new glass vial and dried down in a fume hood. Samples were reconstituted in 150 µl EIA buffer (0.1 mm sodium phosphate buffer, pH 7.0, containing 9 g of NaCl and 1 g of bovine serum albumin per litre) for a 5-fold concentration for lynx hair E, T and P and domestic cat P, and a 10-fold concentration for domestic cat E and T. Reconstituted samples were sonicated for 20 s in an Elmasonic waterbath (Elma GmbH & Co. KG, Germany) before hormone analysis.

Estrogen, testosterone and progesterone values were quantified using EIAs based on the protocols from C. Munro, UC Davis, and described in detail by Kummrow *et al.* (2011). The following antibodies were used: estradiol-17β R4972/R0008, pregnane CL425 and testosterone R156/R157 (C. Munro, UC Davis). In brief, microtitre plates were coated with 50 µl of antiserum diluted in coating buffer (50 mm bicarbonate buffer, pH 9.6) and incubated overnight at 4°C. Unbound antiserum was washed from coated plates with 0.15 m NaCl solution containing 0.05% Tween 20. Immediately, 50 µl of reconstituted hair extracts, hormone standards and controls diluted in EIA buffer were added in duplicate, followed by 50 µl of horseradish peroxidase conjugate diluted in EIA buffer. Plates were incubated for 2 h at room temperature. Following incubation, the plates were washed, and 100 µl of substrate solution (50 mm citrate, 1.6 mm hydrogen peroxide and 0.4 mm 2,20-azino-di(3-ethylbenzthiazoline sulfonic acid) diammonium salt, pH 4.0) was added. Absorbance was measured at 405 nm using a spectrophotometer (MRX microplate reader, Dynex Technologies, Chantilly, VA, USA). Hormone levels are presented as mass per gram dry weight.

### Statistical analysis

Linear regression analysis was used to determine whether there was a significant relationship between the standard curve and serial dilutions for assay parallelism and hormone added vs. hormone recovered for assay accuracy.

Hair reproductive hormones (E, T and P) were used to determine whether differences in reproductive states (intact, estrus, pregnant and spayed/neutered) could be detected, using domestic cats as a model species, given that considerable reproductive hormone data exist for this species (Swanson *et al.*, 1995; Brown *et al.*, 1996; Brown, 2006). Females given oral progestin treatments were excluded from the analysis. Hormones were log transformed to achieve normality and homogeneity of variance. Comparisons were made using a one-way ANOVA and Tukey's *post hoc* HSD test. Given unequal sample sizes, each model was validated further using residual plots. *Post hoc* power analyses were completed using the R package ‘stats: anova.power.test’ (R Core Team, 2013). While there are limitations to the use of *post hoc* power analysis (Hoenig and Heisey, 2001), this method was used because we were unable to conduct *a priori* estimations of sample size due to opportunistic domestic cat sample collection.

Hair reproductive hormones (E, T and P) were used to determine whether differences in age could be detected, because juveniles generally produce lower levels of reproductive hormones than adults, particularly among males (Rolland *et al.*, 2005; Fanson *et al.*, 2010a). Hormones were log transformed to achieve normality and homogeneity of variance. Differences in E, T and P were compared across age (adult vs. juvenile), sex (male vs. female) and region (east vs. west), using a general linear fixed-effect model, from log-transformed estimates. While our focal interest was to differentiate between ages, we included sex in the analysis to assess interaction effects between age and sex.

Hair reproductive hormone ratios (T:E, T:P and E:P) were used to determine whether differences in sex could be detected, given that both sexes can produce testosterone, progesterone and estrogen (Rolland *et al.*, 2005). Ratios were compared across sex, age and region using a general linear fixed-effect model. Hair reproductive hormone ratios were normally distributed (Shapiro–Wilk test, *P* > 0.05). Variances were homogeneous in all cases (Levene's test, *P* > 0.05), except with age and T:P ratio (*F*_1,67_ = 4.22, *P* = 0.044). However, given that this *P*-value is close to the threshold for determining homogeneity (*P* = 0.05), a parametric test was used.

The area under the curve (AUC) of a receiver operating characteristic (ROC) curve (Greiner *et al.*, 2000) was used to quantify how accurately hair reproductive hormone ratios differentiated between sexes, taking into account the number of false positives (i.e. a known male is identified as female, based on hormone ratios). Receiver operating characteristic curves are built by calculating the sensitivity (true positive) and specificity (false negative) at various cut-off points for the data (Boyce *et al.*, 2002); therefore, a plot with perfect discrimination would have an AUC of one, given it has a sensitivity of one and specificity of zero (Greiner *et al.*, 2000). This method is commonly used in the medical and veterinary literature to determine the accuracy of disease diagnosis tests, using the number of true positives and false positives in the test (Greiner *et al.*, 2000). In ecology, ROCs are used to determine the model performance of resource selection functions, using the probability that an animal is detected where it genuinely occurs (true positive) and detection where no animal is observed (false positive; Boyce *et al.*, 2002). In general, AUC values are categorized as follows: 0.5 is considered to be non-informative; 0.7–0.9 is moderately accurate; and >0.9 is highly accurate at discriminating between groups of interest (Swets, 1988; Greiner *et al.*, 2000; Boyce *et al.*, 2002). The R package ‘pROC’ was used to calculate AUC values (Robin *et al.*, 2012).

All statistical analyses were performed using R 2.15.1 (Foundation of Open Source Statistics, Boston, MA, USA).

## Results

### Assay validation: domestic cat

Extraction of exogenous E, T and P in domestic cat hair samples resulted in procedural efficiencies averaging 99.7 ± 9.8 (±SEM), 110.7 ± 3.8 and 90.3 ± 6.8%, respectively. The recoveries of known concentrations of E, T and P from domestic cat hair extracts were as follows: 85.8 ± 4.3, 76.4 ± 2.8 and 100.9 ± 3.8%, respectively. The measured hormone concentrations in the spiked samples correlated with the expected concentrations for all hormones (for E, T and P, *r* = 0.98; all *P* > 0.01; see Supplementary Data). Serial dilutions of pooled hair extracts showed parallel displacement with the standard curves (E, *r* = 0.99; T, *r* = 0.99; and P, *r* = 0.98; all *P* > 0.01; see Supplementary Data). Interassay coefficients of variation were 6.0, 7.4 and 13.0% at 30% binding for E, T and P, respectively, 6.5 and 7.3% at 50% binding for E and P, and 10.4% at 70% binding for T. Intra-assay coefficients of variation were 10.1 at 70% binding for E, and 5.9 and 12.1% at 50% binding for T and P, respectively.

### Assay validation: Canada lynx

Extraction of exogenous E, T and P in lynx hair samples resulted in procedural efficiencies averaging 106.6 ± 10.7 (±SEM), 113.1 ± 13.1 and 96.8 ± 14.9%, respectively. The recoveries of known concentrations of E, T and P from lynx hair extracts were 84.1 ± 1.5, 89.9 ± 7.1 and 73.9 ± 4.1%, respectively. The measured hormone concentrations in the spiked samples correlated with the expected concentrations for all hormones (E, T and P, *r* = 0.99; all *P* > 0.01; see Supplementary Data). Serial dilutions of pooled hair extracts showed parallel displacement with the standard curves (E, *r* = 0.99; T, *r* = 0.98; and P, *r* = 0.99; all *P* > 0.01; see Supplementary Data).

### Reproductive states: domestic cat

**Figure 1:**
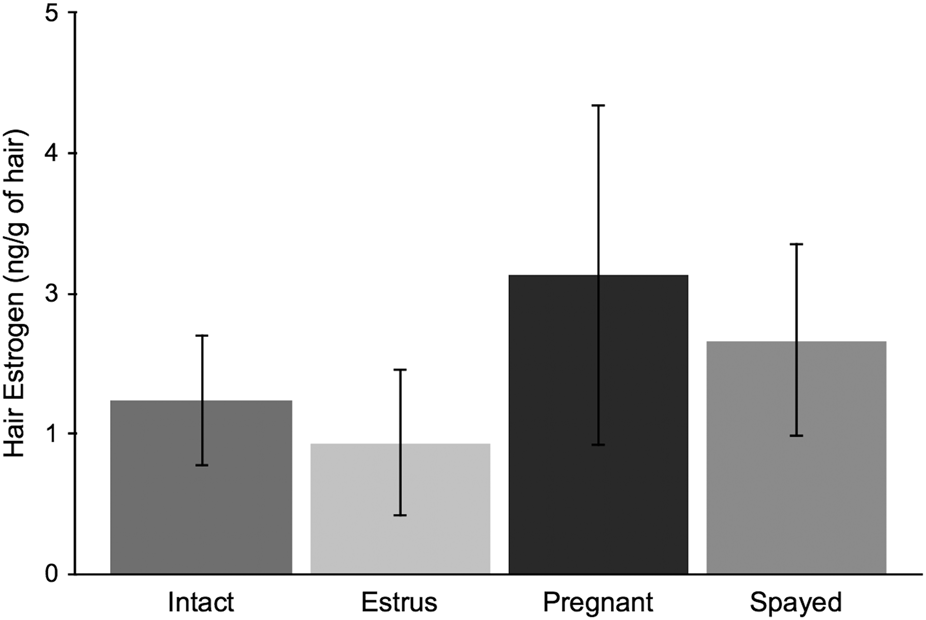
Mean ± SD hair estrogen concentrations (in nanograms per gram of hair) from female domestic cats of known reproductive status (intact, *n* = 4; estrus, *n* =5; pregnant, *n* = 7; and spayed, *n* = 10).

**Figure 2:**
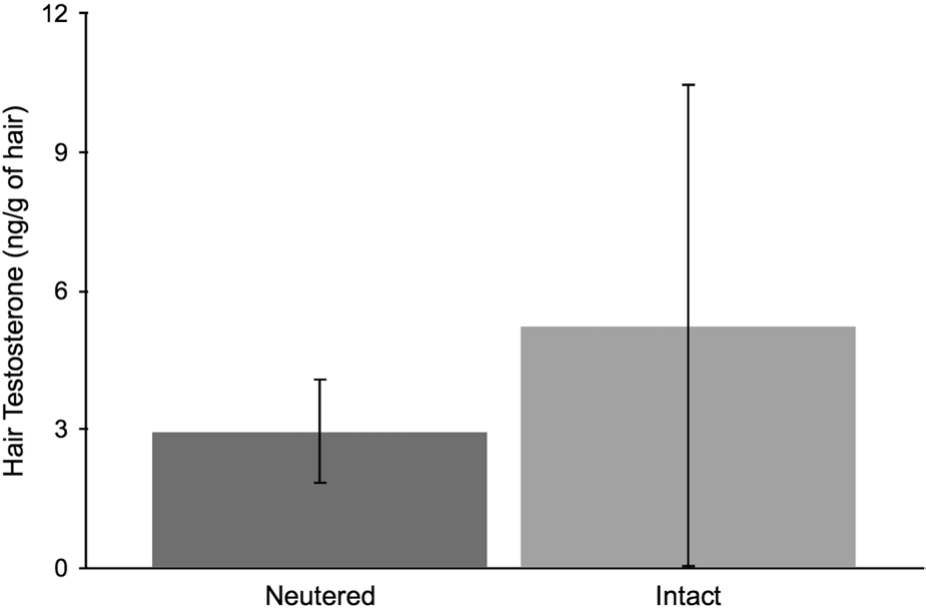
Mean ± SD hair testosterone concentrations (in nanograms per gram of hair) from male domestic cats of known reproductive status (neutered, *n* = 7; and intact, *n* = 5).

**Figure 3:**
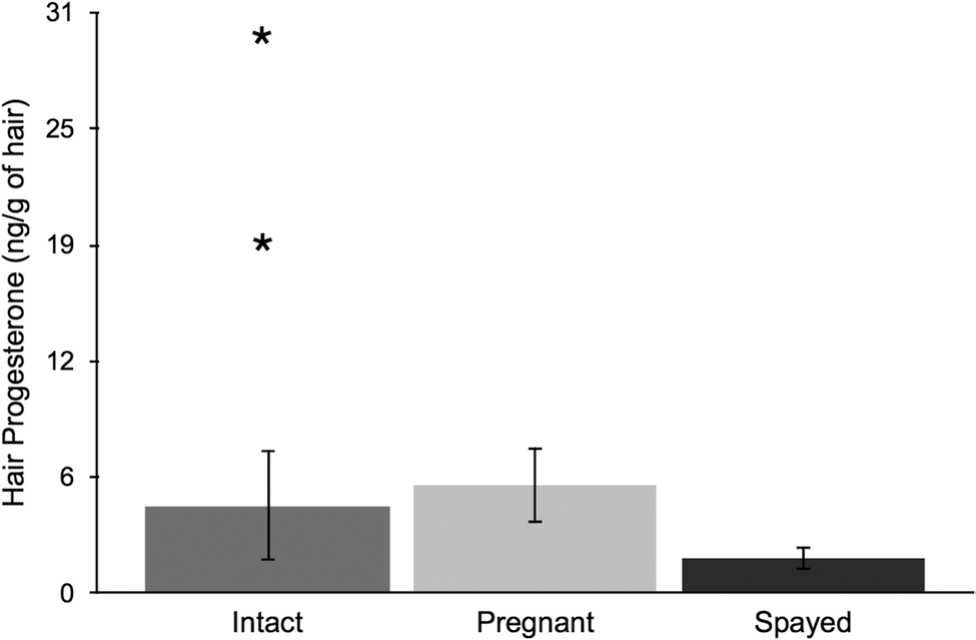
Mean ± SD hair progesterone concentrations (in nanograms per gram of hair) from female domestic cats of known reproductive status (intact, *n* = 10; pregnant, *n* = 5; and spayed, *n* = 10). The asterisks show the hair progesterone concentrations for two females that received a 38 day oral progestin treatment.

Comparisons of hair hormone concentrations for domestic cats of known reproductive states are given in Figs 1–3. Hair E (intact, *n* = 4; estrus, *n* = 5; pregnant, *n* = 7; and spayed, *n* = 10) and T levels (intact, *n* = 5; and neutered, *n* = 7) did not differ significantly between known reproductive states (all *P* > 0.15). Female domestic cat (intact, *n* = 10; pregnant, *n* = 5; and spayed, *n* = 10) hair P levels differed according to reproductive states (*F*_2,24_ * = *9.05, *P* < 0.01). Pregnant individuals had significantly higher P levels than spayed individuals (Tukey's HSD, *P* < 0.01). However, there was no difference between pregnant and intact or spayed and intact individuals (all *P* > 0.08). Incidentally, two female cats were given oral progestin for 38 days ∼6 months prior to analysis, and had discernably higher hair progesterone values (2.7 and 4.13 times the average).

*Post hoc* power analysis indicated that E and P had sufficient power with a sample size of seven individuals per group (E = 0.96, P = 0.98), while T had low power (0.47). In order to achieve a power of 0.8 for T, a sample size of ∼9.5 per group (intact and neutered) would be necessary.

### Age and sex: lynx pelts

Mean hair hormone values for age (adult vs. juvenile), sex (male vs. female) and region (east vs. west) of lynx are given in Table 1. Hair E levels differed between region (*F*_1,64_ = 7.55, *P* < 0.01) and age (*F*_1,64_ = 7.06, *P* < 0.01), but main effects of sex and age × sex were not significant (all *P* > 0.19). These hair E results were supported by AUC values, which revealed a poor ability to classify according to age (AUC, 0.62).

**Table 1:** Mean values ± SD (*n*) of hair reproductive hormones (in nanograms per gram of hair) in lynx pelts according to age, sex and trap line region

	Estrogen	Testosterone	Progesterone
Adult	3.32 ± 1.82 (45)	3.35 ± 1.65 (45)	4.10 ± 2.56 (44)
Female	3.11 ± 1.50 (23)	3.41 ± 1.69 (23)	5.37 ± 2.86 (23)
East	3.02 ± 1.63 (16)	3.16 ± 1.88 (16)	4.81 ± 3.05 (16)
West	3.32 ± 1.26 (7)	3.96 ± 1.07 (7)	6.63 ± 2.01 (7)
Male	3.53 ± 2.11 (22)	3.30 ± 1.65 (22)	2.72 ± 1.12 (21)
East	2.83 ± 1.68 (16)	2.75 ± 1.15 (16)	2.42 ± 0.99 (16)
West	5.43 ± 2.08 (6)	4.76 ± 1.98 (6)	3.69 ± 1.02 (5)
Juvenile	4.34 ± 2.66 (24)	4.06 ± 1.59 (25)	3.93 ± 1.36 (28)
Female	3.85 ± 3.09 (12)	4.04 ± 1.96 (11)	3.89 ± 1.44 (14)
East	3.01 ± 1.09 (11)	3.68 ± 1.63 (10)	3.71 ± 1.32 (13)
West	13.08 (1)	7.67 (1)	6.27 (1)
Male	4.84 ± 2.17 (12)	4.08 ± 1.32 (14)	3.96 ± 1.33 (14)
East	4.79 ± 2.27 (11)	4.03 ± 1.36 (13)	3.94 ± 1.38 (13)
West	5.38 (1)	4.70 (1)	4.27 (1)

Data are not log transformed.

Lynx hair T levels differed between region (*F*_1,65_ = 8.96, *P* < 0.01) and age (*F*_1,65_ = 8.67, *P* < 0.01). However, the hair T values were again not accurate at differentiating age (AUC, 0.65). Differences in hair T were not detected between sex, and no age × sex interaction was found (all *P* > 0.61).

**Figure 4:**
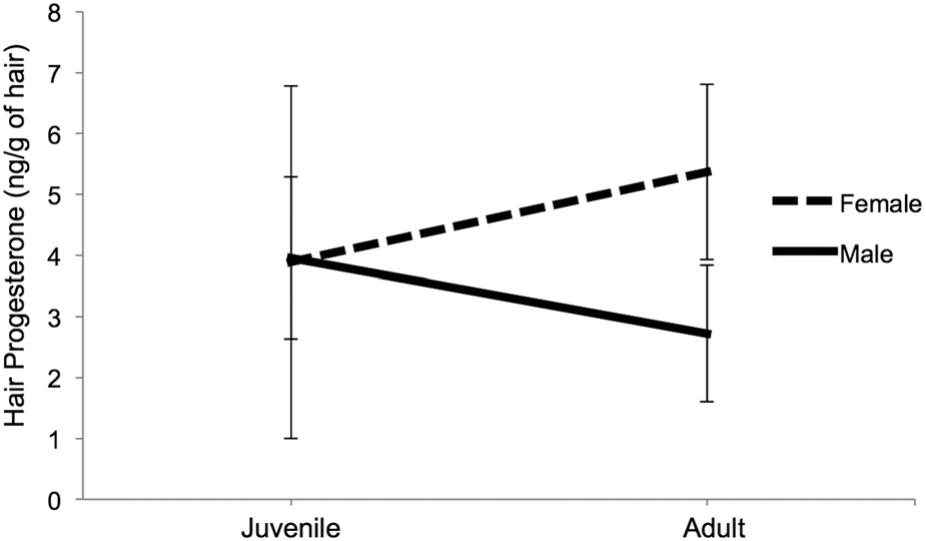
Interaction plot representing differential mean progesterone concentration (in nanograms per gram of hair) according to age class in Canada lynx. Error bars represent ±SD.

Analysis of hair P levels revealed significant main effects of region (*F*_1,67_* = * 12.40, *P* < 0.01) and sex (*F*_1,67_ = 16.04, *P* < 0.01) and an age × sex interaction (*F*_1,67_ = 11.54, *P* < 0.01). However, lynx P levels did not differ between age groups (*P* = 0.14). The significant age × sex interaction term indicates that adult females had higher average hair P values than adult males, while juvenile males and females were not distinguishable (Fig. 4). The AUC values showed that hair P values were non-informative for categorizing age in lynx (AUC, 0.55), although they were moderately accurate at differentiating sex (AUC, 0.70).

Hormone ratios were calculated to determine whether hormone profiles could be used to determine sex using lynx individuals of known sex. Mean hair hormone ratios for age, sex and region are given in Table 2. The T:E ratios did not differ between region or age, and no sex × age interaction was found (all *P* > 0.18); however, they did differ between sex (*F*_1,61_ = 4.82, *P* = 0.03). The AUC values showed that the T:E ratios were not accurate at differentiating between males and females (AUC, 0.65).

**Table 2:** Mean ± SD (*n*) of hair reproductive hormone ratios (in nanograms per gram of hair) in lynx pelts according to age, sex and trap line region

	T:E	T:P	E:P
Adult	1.08 ± 0.25 (45)	0.95 ± 0.39 (44)	0.92 ± 0.43 (44)
Female	1.15 ± 0.28 (23)	0.73 ± 0.37 (23)	0.67 ± 0.39 (23)
East	1.10 ± 0.29 (16)	0.74 ± 0.35 (16)	0.68 ± 0.25 (16)
West	1.24 ± 0.23 (7)	0.70 ± 0.44 (7)	0.64 ± 0.62 (7)
Male	1.01 ± 0.20 (22)	1.19 ± 0.25 (21)	1.20 ± 0.28 (21)
East	1.06 ± 0.21 (16)	1.18 ± 0.25 (16)	1.14 ± 0.25 (16)
West	0.88 ± 0.11 (6)	1.23 ± 0.29 (5)	1.38 ± 0.33 (5)
Juvenile	0.99 ± 0.32 (21)	1.03 ± 0.28 (25)	1.11 ± 0.44 (24)
Female	1.08 ± 0.39 (9)	1.00 ± 0.33 (11)	0.99 ± 0.46 (12)
East	1.14 ± 0.37 (8)	0.98 ± 0.33 (10)	0.89 ± 0.32 (11)
West	0.59 (1)	1.22 (1)	2.09 (1)
Male	0.92 ± 0.25 (12)	1.05 ± 0.25 (14)	1.23 ± 0.40 (12)
East	0.93 ± 0.26 (11)	1.05 ± 0.26 (13)	1.23 ± 0.42 (11)
West	0.87 (1)	1.10 (1)	1.26 (1)

Abbreviations: E, estrogen, P, progesterone; and T, testosterone. Data are not log transformed.

**Figure 5:**
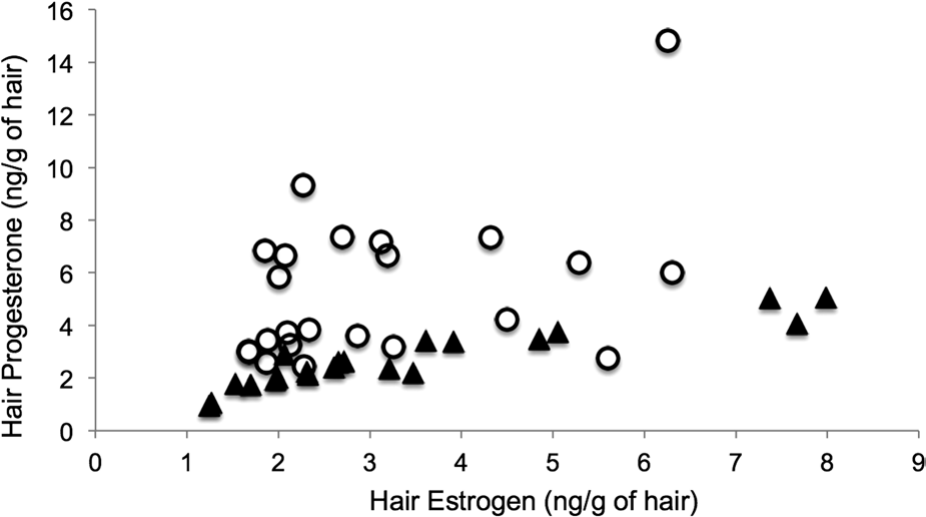
Relationship between progesterone and estrogen (in nanograms per gram of hair) for wild adult Canada lynx pelts of known sex. Open circles, female; and filled triangles, male.

Lynx hormone E:P ratios showed detectable differences between sex (*F*_1,63_ = 23.34, *P* < 0.01) and age (*F*_1,63_ = 5.02, *P* = 0.02), while there were no differences between region, and no sex × age interaction was found (*P* > 0.11). The 95% confidence intervals for adult E:P ratios were non-overlapping, indicating they can be used to distinguish sex (males, 95% confidence interval = 1.08–1.32; females, 95% confidence interval = 0.51–0.83; Fig. 5). The T:P ratios were comparable across region and between ages (all *P* > 0.33), while an effect of sex (*F*_1,64_ = 17.80, *P* < 0.01) and a sex × age interaction were detected (*F*_1,64_ = 6.94, *P* = 0.01). Although both E:P and T:P ratios showed differences in sex, further quantifiaction using AUC values showed that E:P ratios (AUC, 0.82) were moderately accurate and superior at differentiating between sexes when compared with T:P ratios (AUC, 0.75). Finally, the level of accuracy was increased for E:P ratios to highly accurate when only adults were used in the ROC analysis (AUC, 0.90).

## Discussion

To our knowledge, this is the first study to validate an EIA for analysis of reproductive hormones (E, T and P) in the hair of a felid species. First, we used domestic cats as a model to determine whether hair reproductive hormones could differentiate between individuals having known reproductive states (intact, pregnant, estrus and spayed/neutered). Progesterone appeared to be the most discriminating hormone, because pregnant individuals had higher levels than spayed individuals. Elevation in progesterone levels is tied to formation of corpora lutea, and this increase persists through pregnancy (Verhage *et al.*, 1976; Swanson *et al.*, 1995). Progesterone also increases in non-pregnant cats, although the duration is half that of pregnant individuals, at 36–38 days (Brown, 2006). This may explain why pregnant individuals had the highest hair progesterone levels, followed by intact and spayed groups. In addition, administration of oral progestin for 38 days resulted in higher than average hair progesterone values in two females. This provides a preliminary pharmacological validation and indicates the potential of the method for detecting change in hormone levels. Faecal studies support this finding, because they have shown that baseline progesterone levels increase in domestic cats when given oral progestin (Stewart *et al.*, 2010).

Estrogen did not show differences according to domestic cat reproductive states, despite having reasonable statistical power. Although studies have shown that domestic cats are polyestrous and experience brief estrogen surges during estrus (Verhage *et al.*, 1976), cycle lengths are relatively short, 3–16 days at 21 day intervals (Brown, 2011), with only small increases (>10-fold) in estrogen above baseline levels (serum, Verhage *et al.*, 1976; faeces, Graham *et al.*, 1995). It is therefore possible that the small magnitude of estrogen surges is not detectable using hair hormone analysis, given that a longer period of several weeks or months is being measured and compared. These inconsistent results suggest that estrogen from hair may not hold strong potential for monitoring reproductive states.

Testosterone did not differ between neutered and intact domestic cats, although previous studies have shown that faecal testosterone values differ between intact and neutered male domestic cats (Brown *et al.*, 1996). However, given our small sample size, it is possible that the detectable effect size was too low in our testosterone analysis. This may be confounded further by the fact that testosterone levels have high interindividual variation in domestic cats (serum, Johnston *et al.*, 1996; faeces, Brown *et al.*, 1996). These results indicate the potential for hair reproductive hormone analysis in other felids. It is important, however, to validate hormone analysis methods on a species-by-species basis, because felids exhibit large differences in reproductive characteristics, such as type of ovulation, seasonality and ovarian responses (Brown, 2006).

Lynx of known age and sex were used to assess the potential of using hair reproductive hormones to differentiate between groups. Although we detected significant age-specific differences among hair estrogen and testosterone values in lynx pelts, further analysis with AUC showed these differences were not accurate at differentiating between adults and juveniles. Notably, progesterone was effective at differentiating only between the sexes of adult individuals. These findings are similar to faecal reproductive hormone studies that show similarities in estrogen and progesterone values among female lynx aged 2–12 years (Fanson *et al.*, 2010b). However, the results are inconsistent with findings that faecal androgen values differed significantly between neutered males, intact juveniles and adult males (Fanson *et al.*, 2010a). Our inability to distinguish between lynx age classes may be related to error in the age-class classification system, which relied on pelt length measurements (Quinn and Gardner, 1984; Slough, 1996). The method is widely used to distinguishing kits from yearlings and adults (Quinn and Gardner, 1984), meaning that peri-pubertal individuals may be incorrectly classified. Regardless, at this point it is not possible to confirm that these assays hold promise for distinguishing hair hormones by age.

Hair hormone values differed between regions (east and west) for the three hormones of interest. This pattern may be due to differences in the temperature during the time of active hair growth (or moult). For example, the average monthly temperatures from available weather stations were −4.68°C for Ontario and −13.41°C for the Yukon in November 2008 (Environment Canada, 2014); such differences in environmental conditions between areas are likely to persist year round. It is possible that this difference leads to differences in fur characteristics that translate to higher reproductive hormone values in the west. However, while the mechanism driving these regional differences is currently unknown, it is an important potential confounding factor that should be investigated further. Depending on the outcome of this investigation, the utility of hair reproductive hormone analysis should be reviewed carefully.

Hormone ratios showed differences that may be used to distinguish accurately between sexes, especially for adults. In particular, the E:P ratio holds promise for evaluating sex ratios, which is consistent with previous findings showing that faecal androgen-to-estrogen ratios can distinguish sexes in right whales (Rolland *et al.*, 2005) and barred owls (Wasser and Hunt, 2005). Ultimately, this method may provide a cost-effective and reasonably accurate method for determining population sex ratios. Interestingly, these results did not show significant regional differences as observed in the E, T and P values.

We were able to detect reproductive hormones in the hair of other lynx species [Eurasian lynx (*Lynx lynx*) and bobcat (*Lynx rufus*)] and biochemically validated an EIA for lynx and domestic cats, which shows that the method holds promise for *Lynx* spp. (C. V. Terwissen, G. F. Mastromonaco and D. L. Murray, unpublished observations), as well as other felids. Our study should serve as a prompt for further investigation into the use of hair analysis for documenting reproductive states. This is particularly important given that felids tend not to reproduce well in captivity (Brown, 2006), making monitoring of wild populations critical. However, we stress that considerable further pharmacological validation should be conducted using methods to determine that biologically relevant changes are being detected (Brown *et al.*, 1998; Axnér *et al.*, 2008). Accordingly, further development and refinement of hair hormone-based tools will be necessary before these tools can be applied broadly to wild populations.

## Supplementary material

Supplementary material is available at *Conservation Physiology* online.

## Funding

This work was supported by the Toronto Zoo and by a Natural Sciences and Engineering Council of Canada Strategic Projects Grant to D.L.M.

## Supplementary Material

Supplementary Data
