# Supplementary material for: Enzyme immunoassays as a method for quantifying hair reproductive hormones in two felid species
Source: Conserv Physiol. 2014 Oct 11;2(1):cou044. doi: 10.1093/conphys/cou044 (PMC4732485; doi:10.1093/conphys/cou044)
Supplement: Supplementary Data [file supp_2_1_cou044__index.html]

Supplementary Data 

# Enzyme immunoassays as a method for quantifying hair reproductive hormones in two felid species

## Supplementary Data

Supplementary Data

**Files in this Data Supplement:**

- Supplementary Figure 1 - jpg file
- Supplementary Figure 2 - jpg file
- Supplementary Figure 3 - jpg file
- Supplementary Figure 4 - jpg file
