# Supplementary figures and images for: Enzyme immunoassays as a method for quantifying hair reproductive hormones in two felid species
Source: Conserv Physiol. 2014 Oct 11;2(1):cou044. doi: 10.1093/conphys/cou044 (PMC4732485; doi:10.1093/conphys/cou044)

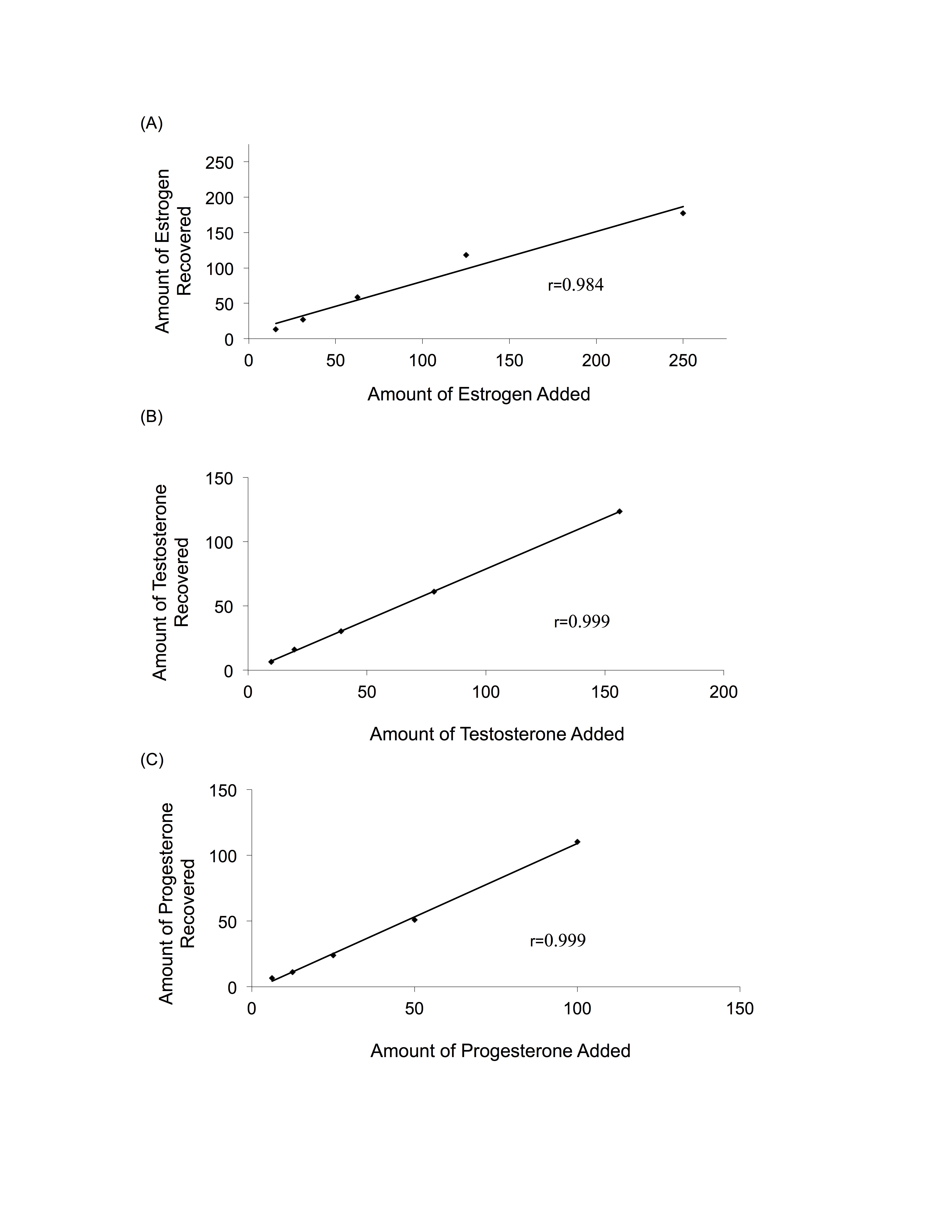

Supplement: Supplementary Data [file supp_cou044_cou044supp_fig1.jpg]

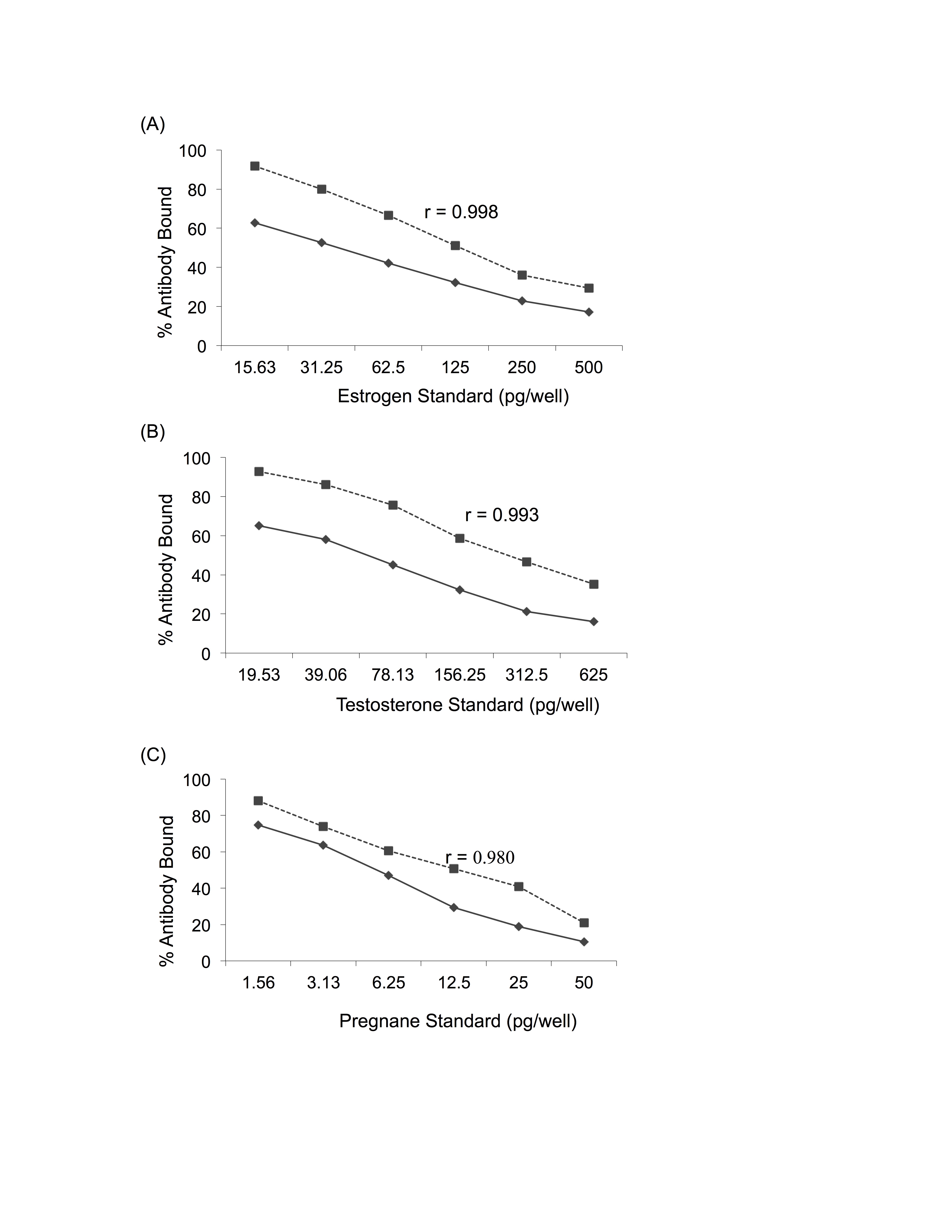

Supplement: Supplementary Data [file supp_cou044_cou044supp_fig2.jpg]

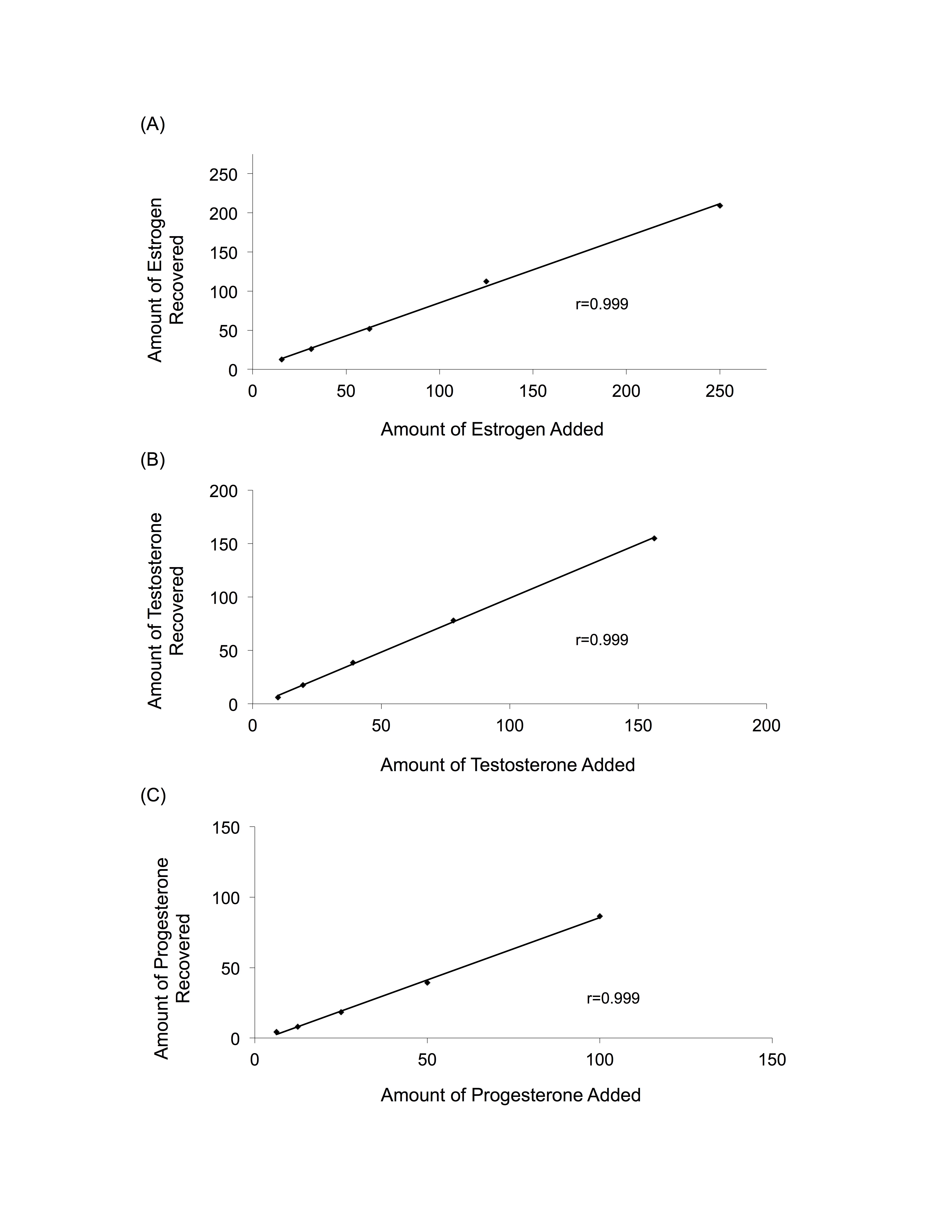

Supplement: Supplementary Data [file supp_cou044_cou044supp_fig3.jpg]

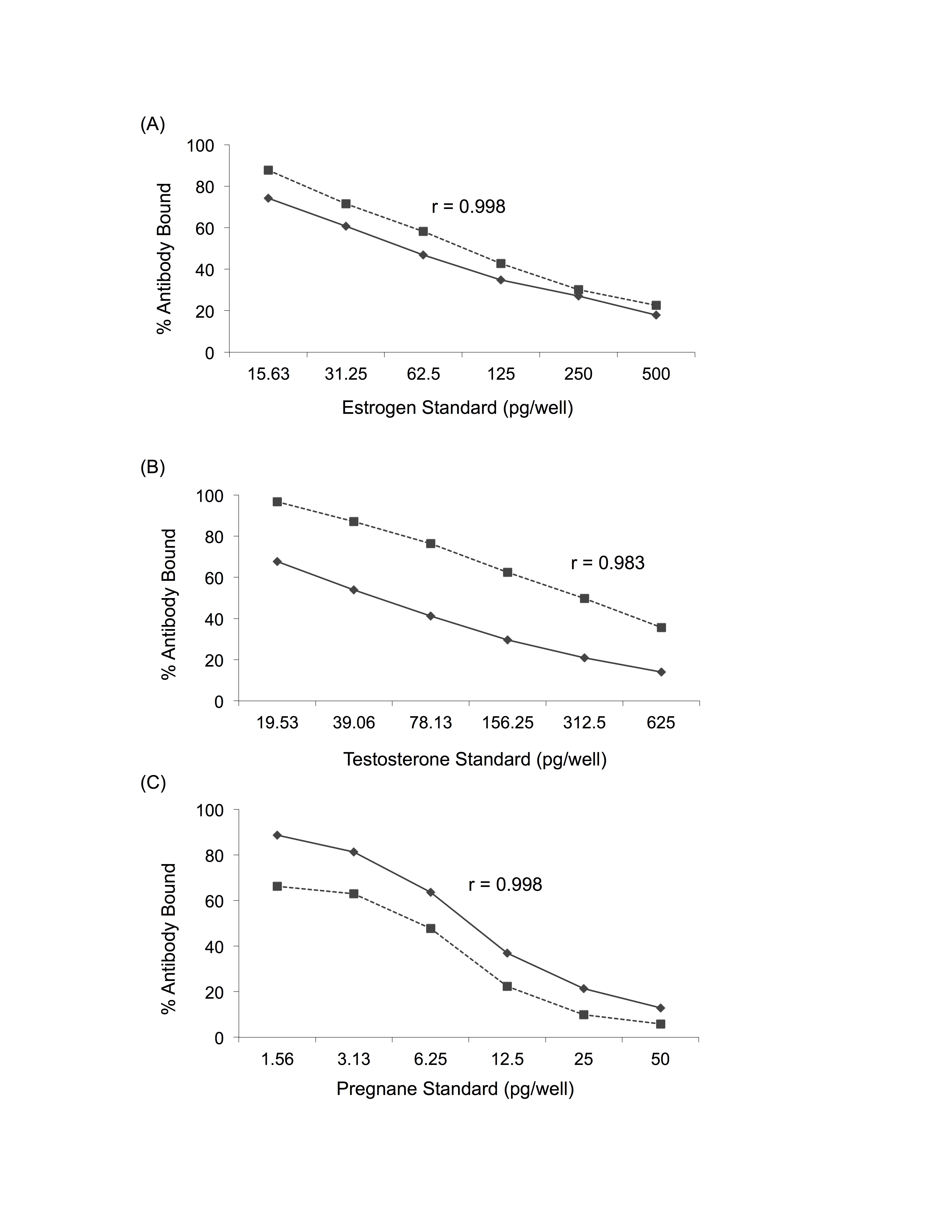

Supplement: Supplementary Data [file supp_cou044_cou044supp_fig4.jpg]
